# Oncological Outcome After Lymph Node Dissection for Cutaneous Squamous Cell Carcinoma

**DOI:** 10.1245/s10434-023-13306-9

**Published:** 2023-03-29

**Authors:** Eva A. Huis in ’t Veld, Thomas Boere, Charlotte L. Zuur, Michel W. Wouters, Alexander C. J. van Akkooi, John B. A. G. Haanen, Marianne B. Crijns, Myles J. Smith, Antien Mooyaart, Marlies Wakkee, Aniel Sewnaik, Dirk C. Strauss, Dirk J. Grunhagen, Cornelis Verhoef, Andrew J. Hayes, Winan J. van Houdt

**Affiliations:** 1 Department of Surgical Oncology, Netherlands Cancer Institute, Plesmanlaan 121, 1066CX Amsterdam, The Netherlands; 2 Department of Dermatology, Erasmus MC Cancer Institute, Rotterdam, The Netherlands; 3 Department of Head and Neck Surgery, Netherlands Cancer Institute, Amsterdam, The Netherlands; 4 Department of Biomedical Data Sciences, Leiden University Medical Center, Leiden, The Netherlands; 5 Department of Medical Oncology, Netherlands Cancer Institute, Amsterdam, The Netherlands; 6 Department of Dermatology, Netherlands Cancer Institute, Amsterdam, The Netherlands; 7 Department of Surgical Oncology, Royal Marsden Hospital, London, UK; 8 Department of Pathology, Erasmus MC Cancer Institute, Rotterdam, The Netherlands; 9 Department of Head and Neck Surgery, Erasmus MC Cancer Institute, Rotterdam, The Netherlands; 10 Department of Surgical Oncology, Erasmus MC Cancer Institute, Rotterdam, The Netherlands

## Abstract

**Background:**

Although cutaneous squamous cell carcinoma (cSCC) is common, lymph node metastases are relatively rare and are usually treated with lymph node dissection (LND). The aim of this study was to describe the clinical course and prognosis after LND for cSCC at all anatomical locations.

**Methods:**

A retrospective search at three centres was performed to identify patients with lymph node metastases of cSCC who were treated with LND. Prognostic factors were identified by uni- and multivariable analysis.

**Results:**

A total of 268 patients were identified with a median age of 74. All lymph node metastases were treated with LND, and 65% of the patients received adjuvant radiotherapy. After LND, 35% developed recurrent disease both locoregionally and distantly. Patients with more than one positive lymph node had an increased risk for recurrent disease.

165 (62%) patients died during follow-up of whom 77 (29%) due to cSCC. The 5-year OS- and DSS rate were 36% and 52%, respectively. Disease-specific survival was significantly worse in immunosuppressed patients, patients with primary tumors >2cm and patients with more than one positive lymph node.

**Conclusions:**

This study shows that LND for patients with lymph node metastases of cSCC leads to a 5-year DSS of 52%. After LND, approximately one-third of the patients develop recurrent disease (locoregional and/or distant), which underscores the need for better systemic treatment options for locally advanced cSCC. The size of the primary tumor, more than one positive lymph node, and immunosuppression are independent predictors for risk of recurrence and disease-specific survival after LND for cSCC.

Nonmelanoma skin cancer is (NMSC) the most frequent malignancy, with an increasing incidence among Caucasians worldwide. Approximately 80% of all NMSC are basal cell carcinomas (BCC) and the second-largest group is cutaneous squamous cell carcinomas (cSCC). The incidence rates for cSCC varies between 23 / 100 000 person-years (average rate) and 387 / 100 000 person-years (in Australia).^1–3^

Although the vast majority of patients with cSCC present with localized disease and are successfully cured with a wide local excision,^4^ approximately 4% of all patients develop metastases, mainly located in the lymph nodes (87%) making cSCCs responsible for the most NMSC deaths.^5^ Metastatic cSCC has a poor prognosis compared to localized disease, since overall survival (3-year) diminishes markedly (ranging from 65-68% to 29-46%) once loco‐regional or distant spread has occurred.^6^ Chemotherapy and Epidermal growth factor receptor (EGFR) inhibitors were the most frequently used systemic therapies for metastatic cSCC.^1,7^ However, in recent years several studies have demonstrated the promising effect of immunotherapy in locally advanced and distant metastatic cSCC.^8–10^

For patients with regional lymph node metastases, a lymph node dissection (LND) is considered standard of care and is often combined with postoperative radiotherapy. LND can be done at three basins: neck, axillary and inguinal, depending on the location of the tumor.^11^ Radiotherapy is indicated in case of more than one positive lymph node, a node >3cm and extracapsular spread.^11–13^

However, the current literature on clinical outcome after complete LND in stage III cSCC consists of small studies or is in particular focused on tumors in particular anatomical locations.^14–16^ Therefore, the aim of this study was to describe the clinical course and prognosis after LND in a large cohort including patients with cSCC lymph node metastases at all anatomic locations.

## Material and Methods

### Patients

All patients with pathological confirmed lymph node metastases from cSCC, without previous LND presented in one of three tertiary referral centres for treatment between 1995 and 2018, were retrospectively identified. The three tertiary centres included were the Royal Marsden Hospital (RMH), London, United Kingdom; the Netherlands Cancer Institute Antoni van Leeuwenhoek (AVL), Amsterdam, the Netherlands; and Erasmus MC (EMC) - Cancer Institute, Rotterdam, the Netherlands. Patient, tumor and treatment characteristics as well as outcomes were obtained from patient files. Patient and tumor characteristics were grouped for analysis according to the 8th edition TNM staging of the AJCC for cSCC.

Recurrent disease was defined as in transit- metastases, recurrent lymph node metastases and distant metastases after LND. Patients with metastatic cSCC from an unknown primary tumor, patients who did not undergo resection of the lymph node metastases, and patients with distant metastases before or simultaneously with lymph node metastases were excluded (n=58). Recurrence-free survival (RFS) was defined as time from LND until first relapse event, including in transit-, lymph nodal- or distant metastases. Disease-specific survival (DSS) was defined as time to from LND until death due to cSCC (complications, metastasis or treatment of the cSCC) and overall survival (OS) as time to death from any cause.

The age of the patient was noted as the age at primary cSCC. The length of follow-up was recorded as date of LND until date of last contact (clinic visit or phone call) or death.

This study was approved by the institutional review board.

### Statistical Analysis

IBM SPSS statistics 25 and R (R Core Team, 2019) were used for the statistical analyses. All data was noted as median with interquartile (IQ) range. Categorical differences between immunosuppressed- and immunocompetent patients were assessed by the Chi-squared test. Recurrence rates were calculated using the cumulative incidence curves accounting for competing risk of death. Differences between cumulative incidence curves (CIC) were computed using Fine and Gray’s Test. Survival analysis was performed by the Kaplan Meijer method, and prognostic factors were identified by univariable Cox regression analysis. The following covariates were included: gender, age size, site, thickness, perineural invasion and differentiation grade of the primary tumor, resection margin, immunosuppression, postoperative radiotherapy, extracapsular spread, differentiation grade, size and number of positive lymph nodes. Covariates with a p-value of ≤ 0.1 were subsequently included in a multivariable analysis. A p-value ≤0.05 was noted as significant. Missing data was corrected by multiple imputations in the regression analysis.

## Results

### Patient and Tumor Characteristics

In total, 268 patients were identified with a median age at primary cSCC of 74 years (IQ 65-82). Within the patient population, 28 (10%) patients were immunosuppressed: 13 (5%) suffered concurrent hematologic malignancy, seven patients (3%) had an organ transplantation requiring immunosuppressive medication, two patients (1%) had HIV, and the other six patients (2%) used chronic steroids for autoimmune diseases. The majority of the cSCCs (80%) were located in the head and neck region. All patient and tumor characteristics are depicted in Table 1.

**Table 1:** Baseline patient and tumor chracteristics

	N(%)
*Gender*	
Male	201 (75)
Female	67 (25)
Age in years at primary tumor (median, range)	74 (33-95)
*Immunosuppression*	
No	240 (90)
Yes	28 (10)
*Size primary tumor*	
<2cm	24 (9)
>2cm	90 (34)
Unknown	154 (58)
*Site*	
Head and Neck	215 (80)
Extremity	43 (16)
Trunk	10 (4)
*Resection margin primary tumor*	
R0	159 (59)
R1	55 (21)
Unknown	54 (20)
*Differentiation grade primary tumor*	
Well	25 (9)
Moderately	102 (38)
Poor	63 (24)
Unknown	78 (29)
*Thickness primary tumor*	
<6mm	23 (9)
>6mm	21 (8)
Unknown	224 (84)
*Perineural invasion primary tumor*	
No	61 (23)
Yes	22 (8)
Unknown	185 (69)
*Recurrent primary tumor*	
No	201 (75)
Yes	67 (25)

Data are expressed as *n* (%) unless otherwise specified

All primary tumors were treated with surgery. Twelve patients (5%) received neo-adjuvant treatment with radiotherapy. Adjuvant treatment with radiotherapy (*n*=28) or chemotherapy (*n*=1) was performed in 10% of the primary cSCC. Half of the patients who received adjuvant treatment had positive resection margins (R1).

### Lymph Node Metastases

Of all patients, 71 (26%) presented with lymph node metastases at time of treatment for the primary tumor. For the remaining 197 patients, the median time to lymph node metastases was 10 months (IQ, 5-19). All patients were treated with LND, and 65% (*n*=173) received adjuvant radiotherapy. Median follow-up after LND was 18 months (IQ, 10-38). See Table 2 for characteristics and treatment of lymph node metastases.

**Table 2:** Characteristics and treatment of lymph node metastases

	N (%)
*Differentiation grade lymph node metastases*	
Well	19 (7)
Moderateley	57 (21)
Poor	74 (28)
Unknown	118 (44)
*Number of positive lymph nodes*	
1	72 (27)
>1	120 (45)
Unknown	76 (28)
Number of positive lymph nodes (median, range)	2 (1-32)
*Extracapsular spread*	
No	71 (27)
Yes	94 (35)
Unknown	103 (38)
Size lymph node metastases in mm (median, range)	25 (2-110)
*Additional treatment after LND*	
Radiotherapy	165 (62)
Chemotherapy	6 (2)
Chemoradiation therapy	1 (0.4)
Immunotherapy	1 (0.4)
No additional treatment	97 (36)

Data are expressed as *n* (%) unless otherwise specified

The majority of the patients with lymph node metastases in the head and neck region (n=215) received postoperative radiotherapy (*n*=145, 67%). Extracapsular spread was more common in head and neck patients treated with postoperative radiotherapy (*n*=53, 64%) than in head and neck patients not treated with postoperative radiotherapy (*n*=15, 35%) (*p*=0.002). All other lymph node characteristics (differentiation grade, size and number of positive lymph nodes) did not differ between the patients receiving radiotherapy or not. Of patients with lymph node metastases in the axilla or groin (*n*=53), 21 (40%) received postoperative radiotherapy. No significant difference in lymph node characteristics (extracapsular spread, differentiation grade, size and number of positive lymph nodes) were noted between patients receiving and not receiving postoperative radiotherapy.

The number of positive lymph nodes was significantly higher in immunosuppressed patients (*n*=17/20 (85%) > 1 positive lymph node) compared to non-immunosuppressed patients (*n*=103/172 (60%) > 1 positive lymph node, *p*=0.028). In 76 patients the number of positive lymph nodes was unknown.

### Recurrent Disease After LND

With a median of 6 months (IQ, 4–12) after LND, a total of 94 (35%) patients developed recurrent disease, respectively as lymph node (*n*=33, 12%), in-transit (*n*=30, 11%) and distant metastases (*n*=34, 13%). Three patients developed a combination of distant-, regional-, and in transit metastases. Patients with recurrent lymph node metastases were mostly treated with radiotherapy (*n*=10, 30%) followed by resection of the recurrent metastases in combination with radiotherapy (*n*=9, 27%), resection of the recurrent metastases without radiotherapy (*n*=2, 6%), debulking surgery in combination with radiotherapy (*n*=2, 6%) and chemotherapy (*n*=2, 6%). Eight patient (24%) received best supportive care. Most patients with in-transit metastases were treated with radiotherapy (*n*=10, 33%), followed by an excision in combination with radiotherapy (*n*=8, 27%), immunotherapy (*n*=1, 3%) or chemotherapy (*n*=1, 3%). Four patients (13%) were treated with an excision only and two patients (7%) with isolated limb perfusion. Four patients (13%) received best supportive care. Of the patients with distant metastases, 19 (56%) received best supportive care. Seven patients (21%) received palliative radiotherapy, four patients chemotherapy (12%), two patients underwent excision (6%), and chemo-radiation and immunotherapy with cemiplimab were both administered in one (3%) patient. Most patients had pulmonary metastases (*n*=24, 71%) followed by bone metastases (*n*= 10, 29%). Five patients had liver, kidney, or adrenal gland metastases. Four patients had distant metastasis at multiple locations. Of the 94 patients with recurrent disease, 82 (87%) died during follow-up, of whom 63 (67%) due to cSCC. Patients with distant metastasis had the worst prognosis: two patients (6%) were alive at follow-up, and the median OS from time of distant metastases was 1.5 months (IQ, 0–6).

There was no significant difference in the number of lymph node recurrences in the same nodal basin between patient who received postoperative radiotherapy and who did not, both in head and neck group (*n*=17/145 (12%) versus *n*=5/70 (7%), p=0.85) as the non-head and neck group (axilla or groin) (*n*=5/21 (24%) versus *n*=6/32 (19%), p=0.93).

### Recurrence-Free, Disease-Specific and Overall Survival

**Fig. 1:**
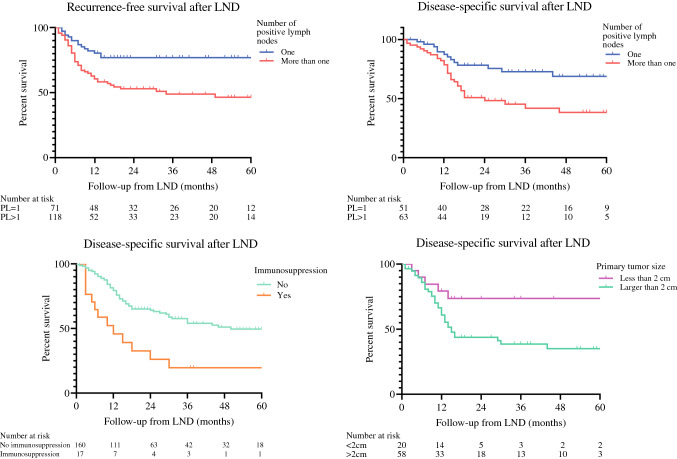
Kaplan Meier curves of recurrence free survival and disease specific survival, patients are classified according to the number of positivie lymph nodes, immunosuppression status and size of the primary tumor. All Kaplan Meier curves are based on non imputated data. LND = lymph node dissection

The 2- and 5-year cumulative incidence for metastatic recurrence after LND was 36% and 38%, respectively. In Univariable analysis, risk for recurrent disease after LND was higher in patients with younger age (HR, 1.03; *p*=0.014), larger primary tumors (HR, 2.51; *p*=0.025), cSCC of the extremity (HR, 2.02; *p*=0.003), immunosuppressed patients (HR, 1.82; *p*=0.042), no postoperative radiotherapy of the lymph node basin (HR, 1.61; *p*=0.018), increased lymph node size (HR, 1.01; *p*=0.044) and more than one positive lymph node (HR, 2.37; *p*=0.002) (Figure 1). In multivariable analysis, only the number of positive lymph nodes (>1) (HR, 2.15; *p*=0.014) negatively influenced recurrence-free survival (RFS) (Table 3).

**Table 3:** Univariable and Multivariable analysis for recurrent disease

	Univariable analysis		Multivariable analysis		
	HR*	*p*-value	HR*	95% CI**	*p*-value
*Gender*					
Female	Reference				
Male	0.943	0.802			
Age in years at primary diagnosis	0.98	0.014	0.98	0.96-1.00	0.074
*Size*					
<2cm	Reference		Reference		
>2cm	2,505	0.025	2.52	0.97-6.54	0.058
*Site*					
Head and Neck	Reference		Reference		
Extremity	2,015	0.003	1,714	0.75-3.93	0.204
Trunk	0.623	0.509	0.392	0.08-2.06	0.269
*Resection margin*					
R0	Reference				
R1	0.803	0.434			
*Differentiation grade primary tumor*					
Well	Reference				
Intermediate	1,358	0.397			
Poor	1.5	0.285			
*Tickness primary tumor*					
<6mm	Reference		Reference		
>6mm	2,339	0.063	2,462	0.90-6.72	0.08
*Perineural invasion primary tumor*					
No	Reference				
Yes	1,296	0.553			
*Immunosupression*					
No	Reference		Reference		
Yes	1,815	0.042	2,399	0.96-5.98	0.061
*PORT of metastases*					
No	Reference		Reference		
Yes	0.608	0.018	1,053	0.56-1.98	0.322
*Lymph node extracapsular spread*					
No	Reference				
Yes	0.978	0.932			
*Differantiation grade lymph node*					
Well	Reference				
Moderate	0.762	0.522			
Poor	1,702	0.167			
Size largest lymph node	1.01	0.044	1,008	0.99-1.02	0.213
*Positive lymph nodes*					
1	Reference				
>1	2,365	0.002	2,147	1.17-3.94	0.014

*HR= Hazard ration. ** CI = confidence interval

Of all patients, 165 (61.6%) died during follow-up, of whom 77 (29%) due to cSCC. The median overall survival (OS) and disease-specific survival (DSS) were 27 (IQ, 11-99) and 172 months (IQ, 14-NR). The 1-, 2- and 5-year OS rates were 70%, 52% and 36%, and the 1-, 2- and 5-year DSS rates were 79%, 65% and 52%, respectively. When patients presented with concurrent lymph node metastases at time of the primary tumor, the DSS was significantly lower than patients who developed lymph node metastases after the primary tumor with a median of 21 months versus 99 months, respectively (*p*=0.02). In univariable analysis, DSS was significantly worse in patients with younger age (HR, 1.03; *p*=0.041), a primary tumor larger than 2cm (HR, 2.60; *p*=0.026) (Figure 1), immunosuppressive- disease or medication (HR, 2.57; *p*=0.002 (Figure 1), who did not undergo postoperative radiotherapy after LND (HR, 2.09; *p*=0.001), and more than one involved lymph node (HR, 2.33; *p*=0.006) (Figure 1). In multivariable analysis, primary tumors larger than 2 cm (HR, 2.59; *p*=0.042), immunosuppression (HR, 3.26, *p*=0.003), and having more than one positive lymph node (HR, 2.14; *p*=0.017) were independent predictors for DSS (Table 4).

**Table 4:** Univariable and Multivariable analysis for disease specific survival

	Univariable analysis		Multivariable analysis		
	HR*	p-value	HR*	95% CI**	p-value
*Gender*					
Female	Reference				
Male	0.873	0.601			
Age in years at primary diagnosis	0.982	0.041	0.984	0.97-1.00	0.094
*Size*					
<2cm	Reference		Reference		
>2cm	2,595	0.026	2,589	1.04-6.48	0.042
*Site*					
Head and Neck	Reference				
Extremity	1,411	0.238			
Trunk	1,636	0.406			
*Resection margin*					
R0	Reference				
R1	1,049	0.869			
*Differentiation grade primary tumor*					
Well	Reference				
Intermediate	0.709	0.341			
Poor	0.815	0.587			
*Thickness primary tumor*					
<6mm	Reference				
>6mm	2,587	0.144			
*Perineural invasion primary tumor*					
No	Reference				
Yes	1,563	0.226			
*Immunosupression*					
No	Reference		Reference		
Yes	2,571	0.002	3,258	1.57-6.78	0.002
*PORT of metastases*					
No	Reference		Reference		
Yes	0.48	0.001	0.687	0.40-1.17	0.165
*Lymph node extracapsular spread*					
No	Reference				
Yes	1,308	0.338			
Size largest lymph node	1.01	0.134			
*Positive lymph nodes*					
1	Reference		Reference		
>1	2,331	0.006	2,141	1.14-4.01	0.017
*Differentation grade lymph node metastases*					
Well	Reference				
Intermediate	0.57	0.218			
Poor	1,252	0.575			

*HR= Hazard ration. ** CI = confidence interval

In immunosuppressed patients, the OS and DSS were 12 (IQ, 5–30) and 15 (IQ, 6–172) months. The 2- and 5-year OS was 30% and 19%, and DSS was 36% and 30%, respectively.

Patients with recurrent metastasis (lymph nodal, distant or local (sub-cutaneous)) had an even worse prognosis with a median DSS and OS for of 15 (IQ, 10–30) and 14 (IQ, 9–27) months from time of LND, respectively. For patients with lymph node recurrences (*n*=32), DSS and OS were 24 months (IQ, 13–44) and 24 months (IQ, 12–36).

## Discussion

This multicentre study analysed the prognosis of 268 patients with lymph node metastases of cSCC who were treated with LND. To the best of our knowledge, this is the largest series describing the course of the disease after lymph node dissection for cSCC at all primary anatomic locations.

This study showed a 5-year DSS and OS of 52% and 36% respectively, while DSS was significantly worse in immunosuppressed patients, patients with tumors >2cm and patients with more than one positive lymph node. The large difference between OS and DSS in this study is likely to be caused by the relatively high age and comorbidities of this patient population. The 2- and 5-year cumulative incidence for recurrence was 36% and 38%, respectively.

When comparing to literature, several smaller studies have described regional lymph node metastasis in the axilla, groin, and head and neck region treated with LND with or without adjuvant radiotherapy.^14–18^ The number of involved lymph nodes is described as a strong independent predictor for RFS, which is in line with the results from our study.^16,17^

For patients with lymph node metastases in the head-neck area, the 5-year OS and DSS rates ranged from 45 to 50% and 60–72%, respectively.^14,18^ The 5-year OS rates in patients with positive lymph nodes in the axilla and groin varied from 32% to 56% (DSS rates were not reported).^15,16^

The documented 5-year RFS rates in other reports varied from 34 to 77% for head-neck patients^14,18^ to 49% for patients with locoregional metastases in the axilla and groin.^15,16^ The reported survival rates in literature seem slightly better compared to our survival rates at all nodal basins. This may be because our study consists of a highly biased patient population referred to a tertiary centre, which can explain poorer prognosis.

Within our patient population, 77 patients died due to cSCC (28.7%), 34 of these patients had proven distant metastasis. The other patients died with inoperable metastatic SCC in the lymph nodes or inoperable in-transit metastases. Some of the patients were referred to a regional outpatient clinic or received best supportive care at home. Since no further imaging was performed there is no more follow-up regarding the development of distant metastases; some patients could have possibly died of tumor growth in vital structures (for example carotid blow out syndrome), but it could well be the case that most patients eventually developed distant metastases.

Postoperative radiotherapy is conducted for local control and studies have shown that there is no influence on survival in either cSCC or melanoma.^11,19^ The National Comprehensive Cancer Network (NCCN) guideline recommends radiotherapy for more than one positive lymph node or a node >3cm.^11^ In addition, the Dutch guidelines recommend postoperative radiotherapy after LND in case of multiple positive lymph nodes and extracapsular spread.^12^ A review confirms the conclusions of both guidelines stating that postoperative radiotherapy can improve clinical outcomes, especially when multiple nodes are involved and extracapsular growth is noted.^13^ In this study, RFS and DSS were influenced by postoperative radiotherapy in univariable analysis (HR of 0.608 and 0.480, respectively). However, no differences were found in multivariable analysis. This could possibly be explained by selection bias, as extracapsular spread was seen more in head and neck patients receiving postoperative radiotherapy compared to patients not receiving radiotherapy. And although no further differences in tumor characteristics were found between those two groups, physicians might tend to opt for radiotherapy earlier in patients where local control could be a problem in case of recurrence.

This raises the question whether postoperative radiotherapy should be added in all high-risk patients according to the guidelines, or whether it may be omitted in selected patients. The benefits of radiotherapy should be carefully weighed against radiotherapy-related morbidity. Ideally, a prospective randomized clinical trial is needed to evaluate the benefit of postoperative radiotherapy after LND.

None of the included patients in this study were treated with postoperative chemo-radiation after 2010. This is in line with the TROG trial, finding no difference in DSS or OS in patients treated with adjuvant chemo-radiation or radiation alone in high-risk stage III or IVa cSCC of the head and neck.^20^

The relatively poor outcome of patients with lymph node metastases emphasizes the need to consider neo-adjuvant or adjuvant studies for this disease with systemic therapy, in line with the melanoma field,^21,22^ since this might increase survival and decrease distant recurrence rates. Systemic therapy in cSCC was until recently limited to cytotoxic chemotherapeutic regimens or EGFR inhibitors.^7,23–29^ However, with the development of immune checkpoint inhibitors new promising treatment options have opened up for this disease.

Recently, Migden et al demonstrated the effect of immune checkpoint inhibition (ICI) with anti-PD1 monoclonal antibody cemiplimab in patients with irresectable locally advanced or distant metastatic cSCC.^8,9^ The response- and durable disease control rate in the metastatic cohort, including 59 patients, was 47% and 61%, respectively. The estimated probability of OS at 12 months was 81% for metastatic cSCC and 93% for locally advanced cSCC patients.^8,9^ These survival rates are more favorable compared with the results of our study. Cemiplimab was therefore also explored in a neo-adjuvant setting, in a trial consisting of 79 patients with resectable stage II-IVa cSCC. Results of this trial show excellent histopathologic complete and near-complete response rates of 51% and 13%, respectively.^30^ Other anti-PD1 ICI, such as pembrolizumab and nivolumab, have been described in small case series with comparable beneficial results.^31–33^ Therefore, combining LND with (neo-) adjuvant ICI seems to be the way forward and might lead to improved survival in this patient group. Whether neo-adjuvant checkpoint inhibition can ever replace LNDs in the future remains to be seen, however, it would be imaginable that surgery could be de-escalated after a major histopathologic response to ICI, which is currently explored in the melanoma field.^34,35^

This study has some limitations. First, due to the selection of patients with lymph node metastases at a tertiary centre, patients are more likely to have more advanced disease or aggressive tumors. Secondly, due to the selection of three referral centres, some patients were lost to follow-up because patients were referred to a hospital closer to home when the LND was performed or when disease became inoperable. To avoid bias in the missing data due to the retrospective nature of this study, we have conducted multiple imputations to fit the statistical models. Also, we have chosen to combine predictors at different time points (both at time of primary tumor as at time of lymph node metastases) for our multivariable analysis based on the assumption that tumor characteristics of the primary tumor may have prognostic implications independent of the time of lymph node metastases. We are aware of the fact that possible micrometastasis at time of the primary tumor can influence these results, however no large studies are performed investigating the rate of micrometastasis at time of primary tumor. Third, the vast majority of the included patients had tumor located in the head and neck region, so one should be careful generalizing the findings of this study to all anatomic areas, although we did not find any statistical differences between the anatomical locations in this study. Unfortunately, the number of patients treated for localized cSCC without regional metastases during the study period was not available for each centre. Therefore, we were not able to compare these numbers to cSCC with lymph node metastases. Finally, the inclusion period of this study was in a time where checkpoint inhibitors were not available for locally advanced or metastasized patients. In the modern times, an increased number of patients in these settings will be treated with cemiplimab or other checkpoint inhibitors, potentially leading to improved outcome. Despite the limitations, this is the largest study to date describing clinical outcome after LND for cSCC in the pre-checkpoint inhibition era at all anatomical locations.

In summary, this study shows that LND for patients with lymphogenic metastasized cSCC leads to a 2- and 5-year DSS of 65% and 52%, respectively. After LND, approximately one-third of the patients develop recurrent disease, of which 35% in the nodal basin. Finally, size of the primary tumor, the number of positive lymph nodes, and immunosuppression or the use of immunosuppressive drugs can be considered as important independent predictors for outcome in patients undergoing LND for cSCC.
